# Concealed and Unconcealed Motives for Joining the Parent-Teacher Association: Mapping Sentence and Smallest Space Analysis

**DOI:** 10.3389/fpsyg.2018.01705

**Published:** 2018-09-11

**Authors:** Yael Fisher

**Affiliations:** Educational Administration, Graduate Studies, Achva Academic College, Arugot Israel

**Keywords:** Facet Theory, SSA, parental involvement, elementary schools, PTA

## Abstract

**Background and Subject:** A Parent-Teacher Association (PTA) is an organization that enables parents to be involved in their children’s schools. Participation in the PTA is one of many steps parents can take to ensure their involvement. Few if any studies have examined parents’ concealed and unconcealed motives (UCM) for joining the PTA.

**Purpose/Hypotheses:** The main purpose was to identify the structure of the concept Motives for Joining the PTA, so as to enhance our understanding of what motivates Israeli parents to join the PTA. The second purpose was to differentiate between parents’ concealed and UCM.

**Method/Procedure:** A self-report anonymous questionnaire, containing 40 items (30 items about aspects of being a member of a PTA and 10 items for reporting background variables), was administrated. Data were collected from a sample of 155 Israeli parents. The initial data processing stage involved EFA (Exploratory factor analysis) using SPSS software. Stage two was a Smallest Space Analysis (SSA), conducted using the Hebrew University Data Analysis Program (HUDAP).

**Results:** EFA indicated three main factors. The internal consistency of the scores in the entire scale, measured using Cronbach’s alpha coefficient, was 0.89. Data deployment on the SSA map exhibited both a polarized form (an angular form) and a radial form in a Radex configuration. The first layer (the polarized facet) was composed of three major motives related to self-serving altruistic ideological motives/ (SSAIM), self-serving altruistic pedagogical motives (SSAPM), and egoistic motives (EM). The second layer (the radial form) was composed of concealed motives, UCM, and politically correct-driven motives.

## Introduction

Parental involvement in the educational system is a subject that researchers around the world have been exploring for years (Mo and Singh, 2008; Fisher, 2010; Jeynes, 2012; Noy, 2014 ). The worldwide implementation of the Law of Compulsory Education over the last century has changed many of the roles that family and society play in child-rearing. Families were forced to collaborate with the State in child-rearing and education, after centuries during which the family alone was responsible for children’s education (Noy, 2014).

The research literature provides evidence of correlations between students’ academic high achievements and parental involvement, based on discussions between parents and children about the importance of school, studying, and the school experience in general (Ingram et al., 2007). The relationship between academic achievements and parental involvement has been found to be statistically significant (Hoover-Dempsey et al., 2002; Jeynes, 2005, 2007; Mo and Singh, 2008).

Parental involvement has been described in various ways, emphasizing both home, school, and community behaviors (Barton et al., 2004; Smith et al., 2011). However, given that there is no single comprehensive definition of parental involvement at school, clarity should be attained in order to reach a consensus among researchers (Fisher and Friedman, 2009).

One of the ways that parents choose to become involved in schools is through the Parent-Teacher Association (PTAs), which in effect consists of parent committees that work alongside teachers to attain shared goals (Fisher, 2011; Jeynes, 2012). The committee’s role is backed by the Director-General Code By-Law. Participation in school- and class-level committees is one of many school-related voluntary activities through which parents opt to get involved in their children’s school experience.

### Parent Committees

There are countries in the Western world in which the various parental delegations have significant statutory powers. For example, there is the PTA, a voluntary organization that includes parents, teachers and staff, which can be found in various countries, such as Japan, United Kingdom, United States (in the majority of countries, its representatives are officially elected in tandem with the presidential elections, and its representatives are appointed to the board of directors of the schools). Elected parents also have authority over the placement of children with disabilities in schools, in contrast to centralized education systems (such as in Israel, in Netherlands, etc.), wherein parents’ authority is limited, and their main function is to advise (Povey et al., 2016).

### Parent Committees in Israel

Parent committees are common in Israeli schools. In Israel, the guidelines for the educational system are published in the Director-General-Code-By-Law which reflect the principles of the educational and administrative policy of the Ministry of Education. The purpose of these guidelines is to define roles and determine procedures and to regulate work processes in the education system. The system includes two types of directives: Standing orders and temporary orders and notices.

Standing orders have binding legal force. A standing order is valid until a new order is written down that revokes or updates it. A temporary order is valid until the expiry date specified in the order itself, for example until the end of the school year. Each instruction contains an introduction detailing the date of entry into force, who it applies to, whether it is a new provision or an update to a previous instruction, as well as a list of previous instructions on the same subject and related subjects. In addition, the person responsible for each of the guidelines and the ways of communicating with him are specified.

The purpose of messages is to provide the schools and their staff with essential information and to arrange work processes, most of which are valid in the school year in which they were published, and at most up to 2 years from the date of publication.

Although more limited in power than the above-mentioned PTA committees, their scope first increased with the establishment of community schools in the 1970s. In 1996, a Director-General Code By-Law was issued, conveying the Ministry of Education’s policy regarding parental involvement in general and in the schools in specific, and expressing its support of parental involvement, mostly emphasizing the need to create a positive climate, but setting ambiguous boundaries (Director-General Code By-Law, SB / 4; *SD* 4 [a]). In a Code By-Law issued in 2003, the Ministry of Education distinguished between three representative offices that parents could hold in the school:

1. A Homeroom Class Committee – a representative group of parents of students assigned to the same homeroom class is democratically elected for the course of the academic year and remains in office until a new representative group is elected for the next school year.
2. A Central Committee of Parents – a committee composed of one or two representatives from each of the class committees, from which a chairman is elected to the institutional committee.
3. Institutional Parents’ Committee – A group of parents of the Central Parents’ Committee is elected to represent the parents of the entire school at district- and national-level forums.

In addition to these committees, there are other parent organizations that vary between different communities and different populations but are not necessarily affiliated with the schools. Most Parent committees are not authorized to intervene in pedagogic decisions of the school, although according to the State Education Law, parents have some degree of influence on the curriculum (25% of the curriculum, but under additional conditions; Fisher, 2010). Most schools believe that although parents have the right to ensure the welfare of their children, when it comes to pedagogic decisions, parents must rely on the professionalism of the teaching staff (Fisher, 2010; Noy, 2014).

**Table 1:** Factor structure of the MJPTA.

Item no.	Item	Factor I	Factor II	Factor III
**Factor I: (9 items; Eigenvalue = 6.99; Explained Variance: 38%; α = 0.84)**				
27.	Wants to be close to the teaching staff	0.854		
4.	Can promote issues related to one’s own child	0.787		
26.	Wants to be close to the child’s teacher	0.768		
25.	Wants to be close to the principal	0.723		0.226
29.	Feels that he/she is contributing to the social involvement of one’s own child	0.625	0.116	0.131
7.	Becomes part of the school administration	0.557	0.194	0.287
2.	Can express a personal opinion on violence in the child’s class	0.521	0.461	−0.173
6.	Can act against the school principal when there is a disagreement	0.471	0.116	0.228
19.	Can affect the treatment of violent events in the child’s class	0.463	0.411	0.223
**Factor II: (7 items; Eigenvalue = 3.67; Explained Variance: 30.8%; α = 0.83)**				
9.	Gives a personal example to the child		0.729	
8.	Feels a partner in the community		0.704	0.112
3.	Can express a personal opinion on violence in general	0.278	0.686	
10.	Gives a personal example to all the students in the school		0.678	
20.	Can affect the response to violent school events		0.626	0.358
24.	Wants to improve the daily life of all schoolchildren		0.570	0.244
11.	Contributes to a pleasant learning atmosphere for students		0.564	0.304
15.	Helps the school principal		0.548	0.474
22.	Wants to take part in social decisions related to the school		0.313	0.290
**Factor III: (7 items; Eigenvalue = 1.84; Explained Variance: 18.281%; α = 0.82)**				
13.	Can add learning contents that are important for school students		0.456	0.705
12.	Can add learning contents that are important for one’s own child	0.223	0.188	0.703
16.	Can affect the level of school teachers	0.218	0.139	0.669
21.	Wants to take part in the school’s pedagogical decisions (and curricula etc.)			0.657
18.	Can affect the school climate		0.579	0.585
14.	Is linked to the local municipality	0.477		0.507
17.	Can influence the climate of the child’s class	0.387	0.118	0.430

### Altruistic Volunteering, Egoism and Concealment

The definition of volunteerism includes a positive attitude toward the activity in which one chooses to engage and toward the cause it serves. The volunteer activity entails no material reward and the volunteer does not receive payment for the service provided. Volunteers play a vital role in helping local schools accomplish their goals and missions. Yet, little is known about the determinants of volunteering in local schools. Thus, community factors, citizens’ concerns, and personal characteristics are possible determinants of general and school volunteering (Wei-Ning and Chin-Chang, 2017). Concepts such as personal (egoistic, narcissistic) and altruistic goals are significant factors among the numerous motivations for volunteering.

Altruistic behavior, generally described as a selfless behavior that benefits a third party’s welfare, sheds light on the phenomenon of “social solidarity in modern societies” (Wuthnow, 1993, p. 344). Due to its significance in explaining social behavior, altruism is broadly studied in social sciences, especially in contrast to the predominant selfish and self-interested behaviors that are characteristic of modern societies, wherein individuals’ primary focus is on personal achievements and goals (Batson, 2016).

Egoistic behavior is described as a situation in which people regularly prioritize their own individual needs and thoughts, above and beyond those of others; hence, they are likely to ignore the feelings, needs, and perspectives of others (Phillips and Phillips, 2011). Egoism and narcissism are interrelated. Every narcissist has a big ego but not every person with a big ego is a narcissist. The 5th edition of the Diagnostic and Statistical Manual of Mental Disorders (DSM) (American Psychiatric Association, 2013)

defines *narcissism* as a personality disorder belonging to Group B of personality disorders. Its main characteristics are excessive preoccupation with oneself and one’s abilities, accompanied by a lack of empathy and an inability to form any kind of sincere relationship (Millon, 2004; Friedman, 2011).

*Concealment* is the act of concealing, i.e., withdrawing or removing something from observation. It also describes a desire to hide, withdraw, or remove oneself from observation; remaining covered and out of sight.

### Definitional Framework and Research Hypotheses

The main purpose of the study was to consider the definitional framework and identify the structure of the concept *Motives for Joining a PTA*.

The second purpose was to tease out the concealed motives, in addition to the unconcealed ones reported. Few if any studies have dealt with parents’ concealed and unconcealed motives (UCM) for joining a PTA-type framework.

A definitional framework for Facet Theory was provided by Guttman (1959). The strength of Facet Theory is in being a methodological approach that facilitates the conceptualization of phenomena. This approach enables the planning and testing of hypotheses, by using data analysis that emphasizes the relationships between the hypotheses and the results (Levy, 1994; Shye and Elizur, 1994). It makes it possible to form a theory based on empirical findings. A theory, according to Facet Theory, hypothesizes a correspondence between a definitional system for a universe of observations and an aspect of the empirical structure of those observations, and includes a rationale for such a hypothesis (Guttman, 1982, p. 335).

Facet Theory uses the mapping sentence as a key tool. The facets describe the research issues and each facet has a different role. The facets make it possible to rationalize the hypothesis of the research (Guttman, 1959). There are three facets: the first facet describes the population (in this study, parents); the second facet describes the study’s main issues (motives and concealment) and is called the content facet; and the third facet describes the range (level of perception).

In the current research, Smallest Space Analysis (SSA) was used for the applications. SSA is a statistical model that makes it possible to examine the concordance between the mapping sentence and the relationships between the variables. A correlation between variables is represented as a physical distance in a two- or multi-dimensional space. The points in space are closer when correlations between variables are greater. The map actually affords a better understanding of the correlation matrix. Indeed, there is no other statistical approach that offers a better means of interpretation.

**FIGURE 1:**
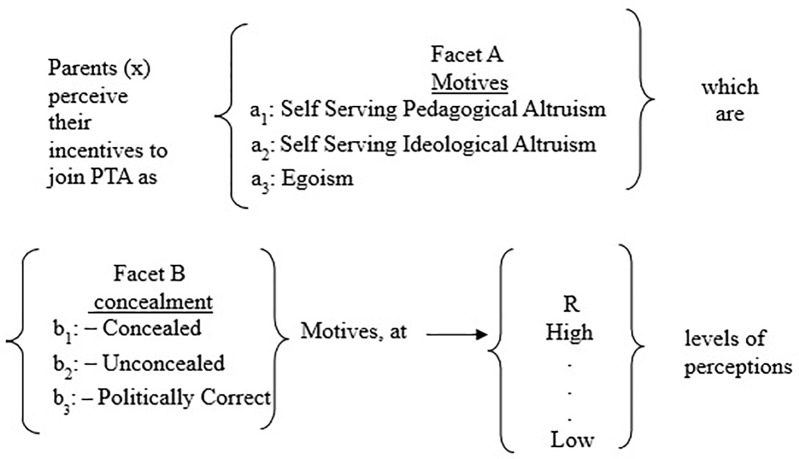
A mapping sentence: perception of motives for joining PTA.

The following mapping sentence (**Figure 1**) describes the parents’ perceptions of their motives for joining a PTA-type committee. The mapping sentence is comprised of two content facets and one range facet:

Facet A, the first content facet (motives) is an unordered facet, since it is not necessary (or possible) to prefer one motive over another. This facet’s role is to deploy the variables in an angular form, creating segments on the SSA map, based on the facet’s elements:

a_1_– Self-Serving Pedagogical Altruism: Parents who act out of pedagogical altruistic motives wish to improve not only the pedagogical contents of their children’s class, but also the pedagogical contents of the school as an organization. They believe that they can contribute to and even improve the knowledge and performance level of school teachers and wish to be influential partners in all of the school’s pedagogical decisions. They also aim to influence their children’s class climate and that of the school, as well. Given that the outcome of their participation is expected to be beneficial to their own children, as well as to others, we refer to these as “self-serving altruistic” motives.
a_2_– Self-Serving Ideological Altruism: Parents who volunteer in order to give a personal example of good citizenship to their children and to all the other students as well. They want to feel that they are a part of a community in which they are active partners. They want to have the ability to express their opinions on violence in school and believe that their input can help eliminate the problem. As part of their ideology, they espouse social and environmental values which they would like to pass on to their children and to other students as well.
a_3_– Egoism: Parents who volunteer in order to be close to their child’s school principal and teachers while promoting issues related to their own child. They feel that they can act against school principals whenever they do not agree with their opinion.

Facet B, the second content facet (concealment), is a hierarchically ordered facet. In a hierarchically ordered facet, the variables in the SSA map are deployed based on the mapping sentence elements, in a radial configuration forming three circles:

b_1_– Concealed motives: These motives (inner circle) are not spoken out loud. Parents do not wish to admit that they joined the PTA in order to be close to the local authorities. They do not tell others that they joined the PTA in order to become part of the school management. They keep those motives to themselves.
b_2_– UCM: These motives are mainstream motives (middle circle). These are conventional motives such as wanting to improve the school curriculum for the benefit of all the students or to improve the school climate.
b_3_– Politically correct motives: These motives are used in order to avoid being offensive (outer circle). They connote what others would want to hear. Instead of saying “We joined the PTA to be part of the core decision makers,” they use the phrase “We want to partake in the school’s pedagogic decisions.” Given that they present themselves using the politically correct terms, they are found acceptable. Thus, they are savvy, but lack uniqueness.

## Materials and Methods

### Subjects

The study population was comprised of 155 parents whose children were attending elementary school.

a) Gender: 114 (73.5%) women and 41 (26.5%) men.
b) Ages: 82 parents (52.9%) were between the ages of 20 and 40 years old, and 73 parents (47.1%) were over 55 years old.
c) Education: 61 parents (39.4%) had an undergraduate degree; 61 parents (39.4%) had a graduate degree; 4 parents (2.6%) had a Ph.D.; 29 parents (18.7%) declared a different level of education.
d) Living location: 45 parents (29%) resided in the northern part of the country; 50 parents (32.3%) in the central part of the country; 60 parents (38.7%) in the southern part of the country.
e) Members in PTA: 73 parents (47.1%) were PTA members; 82 parents (52.9%) were not PTA members.
f) Employment status: 113 parents (72.9%) were salaried workers; 30 parents (19.4%) were self-employed; 12 parents (7.8%) were unemployed.
g) Number of children: 51 parents (32.9%) 1–2 children; 79 parents (51%) 3 children; 25 parents (16.1%) 4 children or more.
h) School size: 128 parents (82.9%) sent their children to schools attended by 400 to 700 students; 22 parents (14.2%) sent their children to schools attended by 700–1000 students; 5 parents (3.4%) did not provide this information.

### Instruments

The research instrument was a new anonymous self-report questionnaire titled “Class and School PTA,” which includes 30 items about aspects of being a member of a PTA (1–5 on a Likert scale) and 10 items for reporting background variables. Altogether, the questionnaire contained 40 items. The questionnaire was prepared specifically for this study (see **Appendix 1**).

### Procedure

Permission to proceed with the study was granted by the Israeli Ministry of Education on October 1, 2013. The study was conducted in two stages.

(a) Defining variables and terminology, generating scale items, and designing the research instrument.
(b) Administering the research questionnaire: the questionnaire was distributed during parent-teacher meetings of the 2014 school year. Parents were asked to complete the questionnaire and return it a few minutes later. A total of 300 questionnaires were distributed during regional meetings. Of these, 155 questionnaires were completed (51.6% return rate).

## Results

The first stage of the data analysis included computation of means for each item, variance, and item-total correlations. A factor analysis was conducted, based on the mapping sentence. This assisted with confirming the content facets concerning motives for joining PTA. Internal consistency of the scale, measured using Cronbach’s alpha coefficient, for the scores of the entire scale “Motives for Joining PTA” (MJPTA) and for the three subscales (self-serving, altruistic ideological motives –SSAIM; self-serving, altruistic pedagogical motives –SSAPM; and egoistic motives –EM) were 0.89, 0.84, 0.81, 0.85, respectively (see **Table 1**).

**FIGURE 2:**
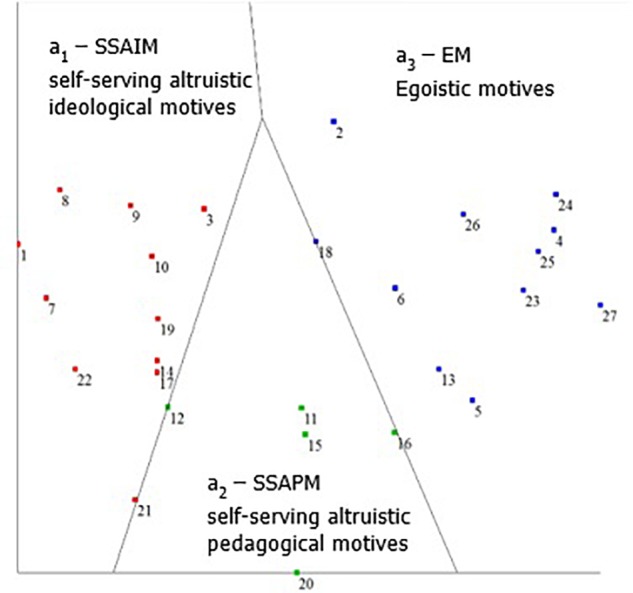
Angular facet A on the SSA map: motives for joining PTA.

**FIGURE 3:**
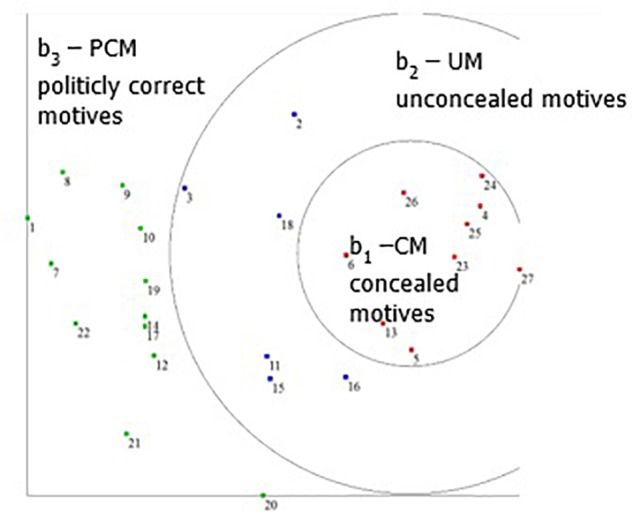
Radial facet A on the SSA map: concealment.

The second level included subjecting the data to an SSA procedure, using the Hebrew University Data Analysis Program (HUDAP; Borg and Shye, 1995), based on the calculated correlation matrix. Data deployment was examined in a two-dimensional space. The results were tested first by applying the coefficient of alienation. The coefficient of alienation was .18, indicating a good match between the initial correlations matrix and the SSA map. Data deployment on the SSA map exhibited both an angular and a radial form, in a Radex configuration. The two deployment patterns, i.e., the angular (**Figure 2**) and the radial (**Figure 3**), are described below.

### The Angular Aspect

The deployment of the variables for facet A (see **Figure 2**) shows that the SSA map has an angular order. The findings demonstrate a perfect fit between the empirical data and the estimated structure (separation index = 1.000). Facet A divides the map into three regions that emanate from a single point, where each region faces a different direction away from that point, as described below. In an angular aspect, items located farthest from the origin point may best express the semantic content of the entire angular region^1^.

The map clearly divided the data into three sections:

(A) The left-hand region of the map contains items which relate to self-serving altruistic ideological motives (SSAIM) (a_1_): “I think that parents join the PTA so that they can express their opinions on school violence in general.” (item 3).
(B) The central region of the map contains items that relate to self-serving altruistic pedagogical motives (a_2_): “I think that parents join the PTA because it contributes to a pleasant learning climate for the students” (item 17).
(C) The right-hand section of the map contains items which relate to EM (a_3_): “I think that a parent joins the PTA to be close to the school principal for his or her own personal benefits” (item 25).

### The Radial Aspect

**Figure 3** shows the radial aspect of the data deployment on the SSA map. The separation index was 1.000, indicating perfect separation. The three circles seen in the data deployment concern the concealment aspect of motives for joining PTA.

As can be seen clearly, the items that are very close to each other are in the inner circle, whereas the items closer to the periphery are more distant from each other, separated by large blank spaces. The proximity of the items in the layout map of the SSA indicates greater consensus and unity of semantic items and terminology. Accordingly, a distance between the items indicates little consensus and greater semantic distinction.

The radial deployment forms a central circle in which the common denominator of all nine items is motives that are not stated openly (b_1_) and points to motives for which there is usually a consensus among parents that do not join PTA. What are the hidden motives of parents that do join? These items are associated with feelings such as gaining personal benefits, being close to the local authorities, or close to the principal and staff. For example: “I think that parents join the PTA to become part of the school administration” (item 7); “I think that parents join the PTA in order to be close to the teachers” (item 26); “I think that parents join the PTA to promote issues related to their own children” (item 4).

In the second concentric circle, namely the middle circle (see **Figure 3**), are positioned motives that are stated openly and thus are unconcealed (b2). These motives are perceived as acceptable motives, mostly mainstream motives that are associated with contributing to the pedagogical and educational contents and to the learning atmosphere. For example: “I think that parents join the PTA because they wish to influence the school climate” (item 18); “I think that parents join the PTA because they wish to influence the treatment of violent events in their children’s classes” (item 19).

The third and outermost circle relates to politically correct motives. These motives are also UCM but are related to what parents think is the proper thing to say to others (a3). Stating politically correct motives is a means to avoid, intentionally or unintentionally, the use of expressions that contain an explicit or implicit allegation, which might be found offensive. For example: “I think that parents join the PTA because they believe that they are contributing to the school community” (item 1); “I think that parents join the PTA because they want to serve as a personal example to their own children” (item 9); “I think that parents join the PTA because they believe that they can help the school principal” (item 15).

## Discussion

This research is unique in that it addresses the issue of parents’ motives for joining the PTA. Although the professional literature has referred to issues related to the PTA framework, it has yet to address the issue of parents’ motives for joining the PTA. To date, the literature has dealt mainly with the roles, responsibilities, and activities of the PTA (Zafar et al., 2013). As we know that joining the PTA is a voluntary activity, we can presume to know the motivations, as they are familiar from the study of volunteerism. Studies in this field have found that this is a type of community involvement that provides positive outcomes, both physical and mental, for both the volunteers and the community (Bhargava and Witherspoon, 2015). The motives can be related to personal, social, moral, or utilitarian aspects (Stukas et al., 2016).

The current findings show that even a complex term, such as “motives for joining the PTA,” can be conceptualized using facets A and B. The hypothesis that was presented in the mapping sentence (see **Figure 1**) is fully supported, meaning that the concept Motives for Joining the PTA (MJPTA) is based on two layers. The first (Facet A) concerns motives related to SSAIM, SSAPM (self-serving altruistic pedagogical motives) and EM. The second layer (Facet B) of the MJPTA concept concerns the concealment of motives: concealed motives (CM), UCM and politically correct motives (PCM).

### Relationships Between Motives

As explained above, the SSA map enables us to understand the relationship between variables that share the same regions, by exploring the distance between them. Each variable is represented by digits or points, so the closer in space they are, the more they share the same content. The other points in the other regions relate to different elements of that facet and the further apart they are, the greater is the difference between the contents they represent (Borg and Shye, 1995).

The order of the facet elements is reflected in the order of the SSA regions, whereas the “role” of the facet in the deployment of the SSA map is reflected in the unique order between the map’s components (Levy, 1994). The higher the correlation is between two items, the closer they are in space and in terms of content.

As can be seen in **Figure 2**, there is a higher correlation (they are closer together) between EM as a group and the SSAIM as a group. Furthermore, most of the motives are perceived either as SSAIM or EM. Observing the circomplex structure, we can see that most of the motives are either concealed motives (CM), such as “They think it will help them in the future to become political activists in their own local community” (item 30) or politically correct motives, such as “Wanting to improve the daily life of all school children” (item 24). It is interesting that the UCM have lower correlations between them (are farther apart).

The professional literature has not yet dealt with the issue of concealed and UCM of parents joining PTA. Therefore, this is an interesting and new finding, that could assist principals, teachers, and policy makers in gaining a better understanding of these phenomena. Understanding these motives, both by teachers and principals, can expand the preferred activities that are based on altruistic motives, since those are the motives that will probably be beneficial to increase student academic achievements (Ingram et al., 2007) and contribution to the school as a better organization (Fisher, 2010). If they are aware of these phenomena, school principals, and teachers can prevent harmful effects from egoistic factors and promote the contributing effects of altruistic factors. Policy makers may also be able to decide when it is right or wrong to involve parents, based on this understanding.

Although this finding has not emerged in previous studies, it may be relevant not only in Israel, but also in other countries. Furthermore, at this point we have no indication of whether the MJPTA concept is culturally based. Therefore, it is definitely important to conduct a cross-national study, in order to expand our knowledge on this most interesting issue. If similar results will be revealed, we will be maybe be able to show that the fundamental concepts presented in this article are not culturally dependent and are rather shared by different cultures and different nations.

## Author Contributions

The author confirms being the sole contributor of this work and approved it for publication.

## Conflict of Interest Statement

The author declares that the research was conducted in the absence of any commercial or financial relationships that could be construed as a potential conflict of interest.

## Footnotes

1 Similar to factor loading in factor analysis: The higher the load, the more relevant it is in defining the factor’s dimensionality (Kim and Mueller, 1978).

## Appendix

**Table A1:** Questionnaire.

	I think that parents join PTA because they:	Strongly disagree	Disagree	Neutral	Agree	Strongly agree
1.	Feel that they are contributing to the school community	1	2	3	4	5
2.	Can express their opinions on violence in their children’s class	1	2	3	4	5
3.	Can express their opinions on violence in school in general	1	2	3	4	5
4.	Can promote issues related to their own children	1	2	3	4	5
5.	Can promote school-related issues	1	2	3	4	5
6.	Can act against the school principal in case of major disagreements	1	2	3	4	5
7.	Become part of the school administration	1	2	3	4	5
8.	Feel they are partners in the community	1	2	3	4	5
9.	Want to serve as a personal example to their own children	1	2	3	4	5
10.	Want to serve as a personal example to all the students in the school	1	2	3	4	5
11.	Believe that they contribute to a pleasant learning atmosphere for students	1	2	3	4	5
12.	Can add learning contents that are important to their own children	1	2	3	4	5
13.	Can add learning contents that are important to school students	1	2	3	4	5
14.	The PTA is linked to the local municipality	1	2	3	4	5
15.	Believe that they can help the school principal	1	2	3	4	5
16.	Can affect the level of school teachers	1	2	3	4	5
17.	Can influence the climate of their own children’s class	1	2	3	4	5
18.	Can affect the school climate	1	2	3	4	5
19.	Can affect the treatment of violent events in their children’s classes	1	2	3	4	5
20.	Can affect the treatment of violence in the school	1	2	3	4	5
21.	Want to take part in the school’s pedagogical decisions (curricula etc.)	1	2	3	4	5
22.	Want to take part in social decisions related to the school	1	2	3	4	5
23.	Want to improve the daily life of their children at school	1	2	3	4	5
24.	Want to improve the daily life of all school children	1	2	3	4	5
25.	Want to be close to the principal	1	2	3	4	5
26.	Want to be close to their children’s teachers	1	2	3	4	5
27.	Want to be close to the teaching staff	1	2	3	4	5
28.	Feel that they are contributing to improving the achievements of their own children	1	2	3	4	5
29.	Feel that they are contributing to the social involvement of their own children	1	2	3	4	5
30.	Want to become political activists in their own local community in the future	1	2	3	4	5
